# Supplementary material for: Lessons Learned from the Lessons Learned in Public Health during the First Years of COVID-19 Pandemic
Source: Int J Environ Res Public Health. 2023 Jan 18;20(3):1785. doi: 10.3390/ijerph20031785 (PMC9914715; doi:10.3390/ijerph20031785)
Supplement: Supplementary file 1 [file ijerph-20-01785-s001.zip › ijerph-2143815-supplementary.pdf]

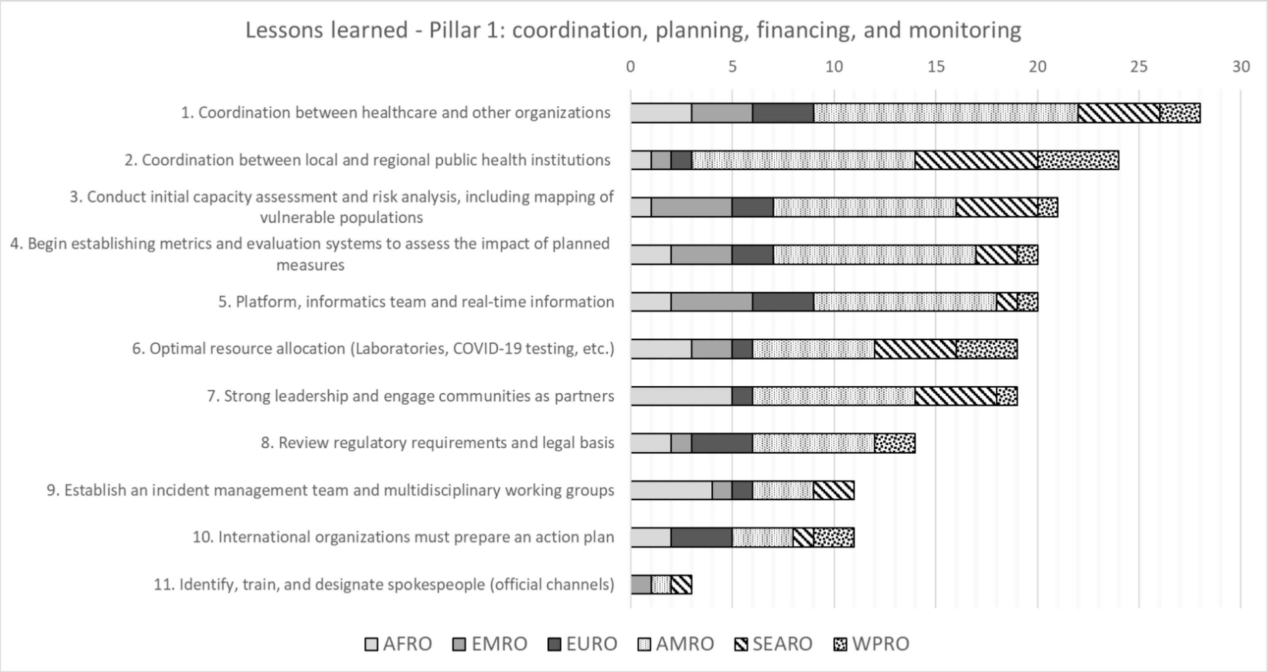

Figure S1. Pillar 1: Ranking of lessons learned and number of their citations in papers from different WHO Regions

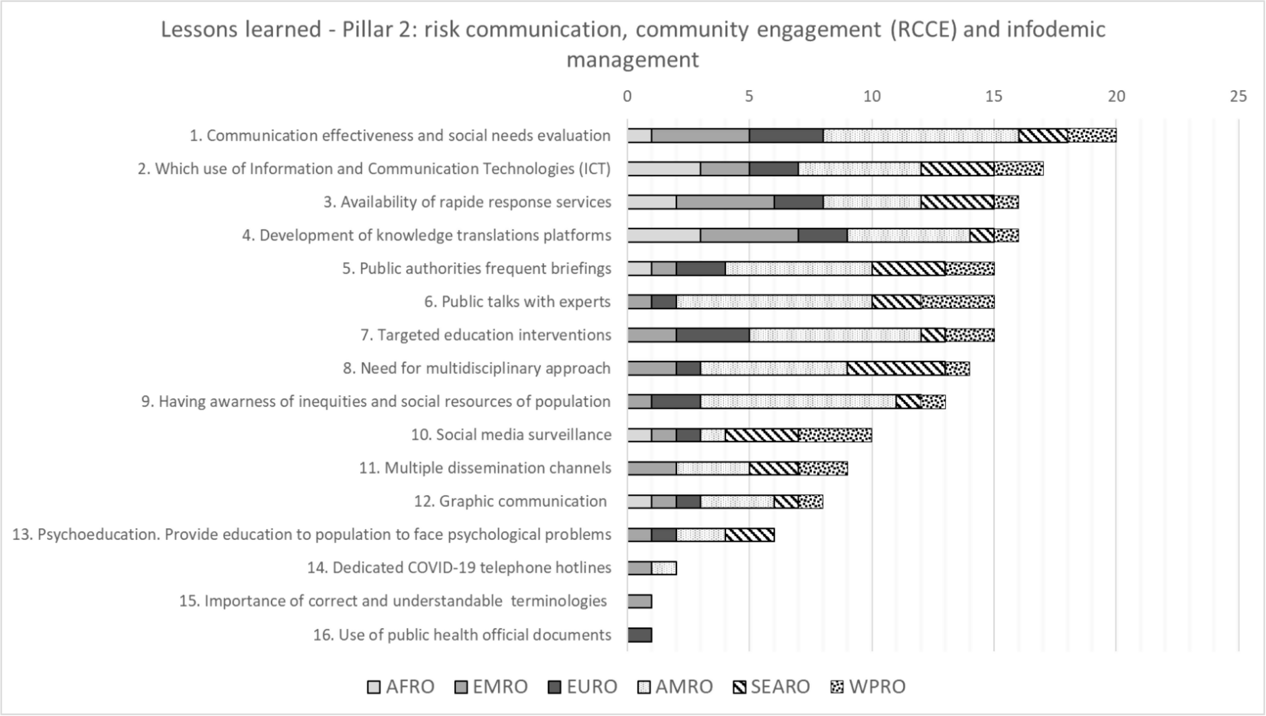

Figure S2. Pillar 2: Ranking of lessons learned and number of their citations in papers from different WHO Regions

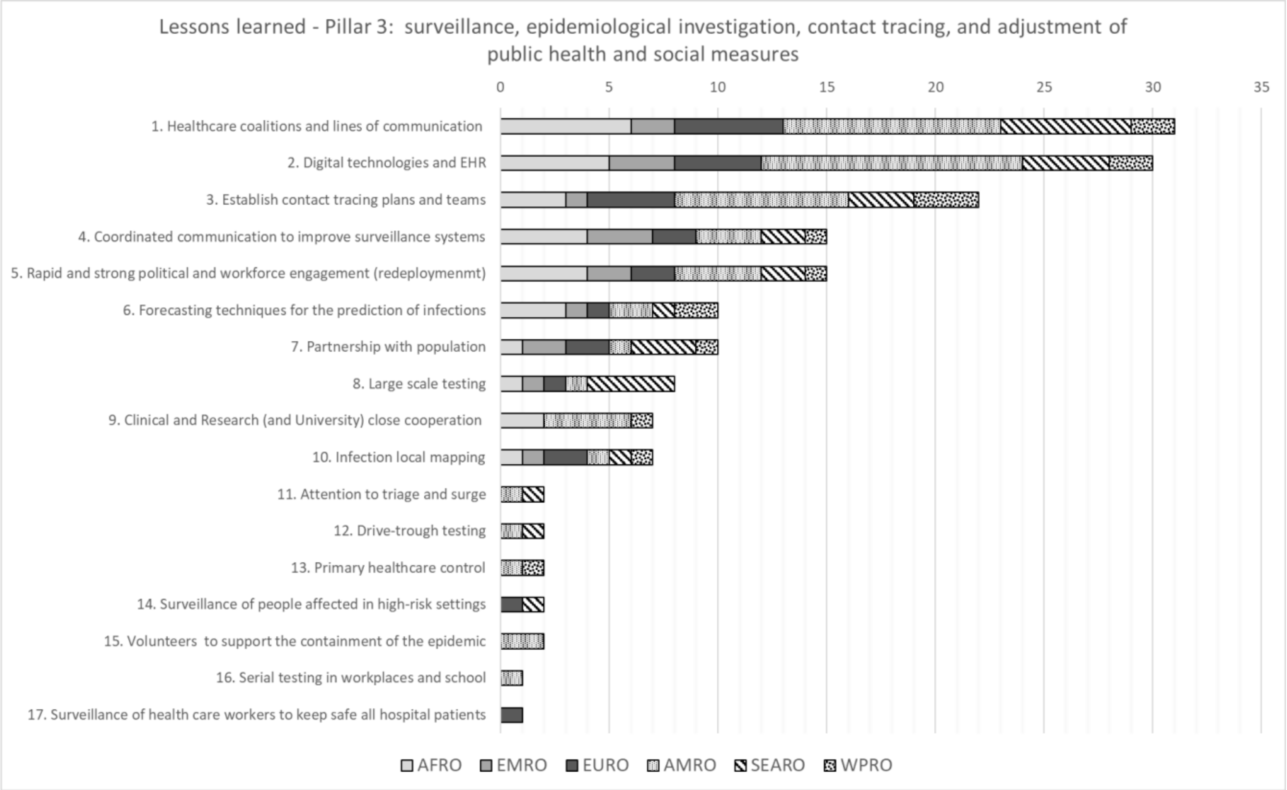

Figure S3. Pillar 3: Ranking of lessons learned and number of their citations in papers from different WHO Regions

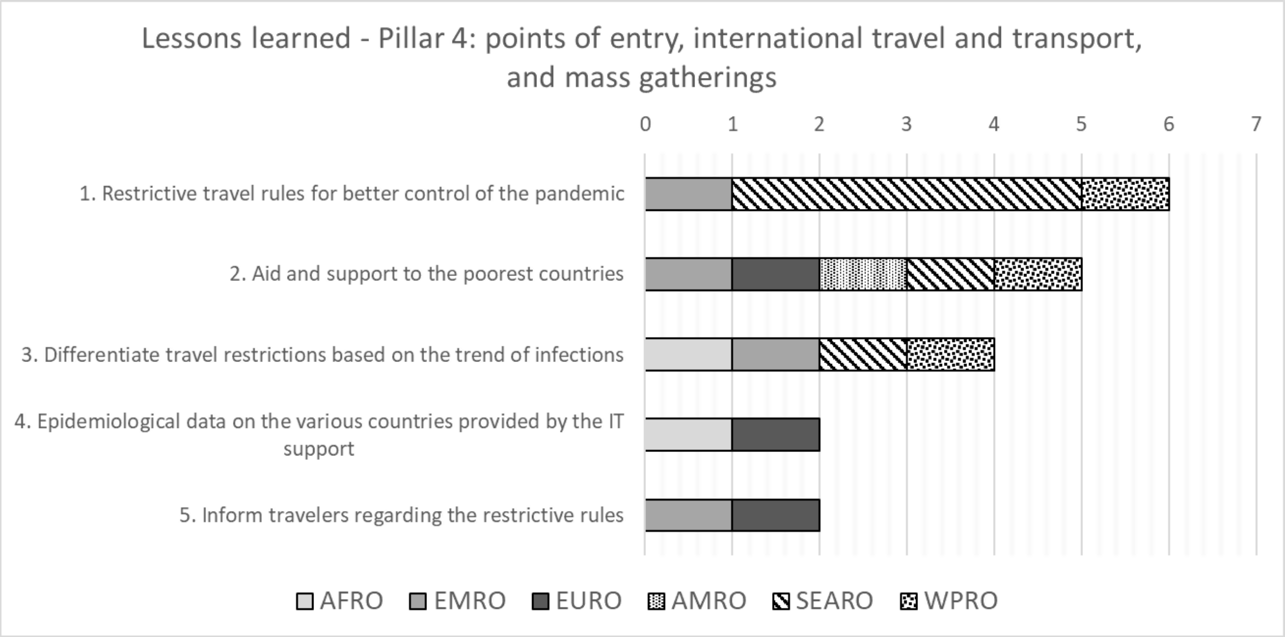

Figure S4. Pillar 4: Ranking of lessons learned and number of their citations in papers from different WHO Regions

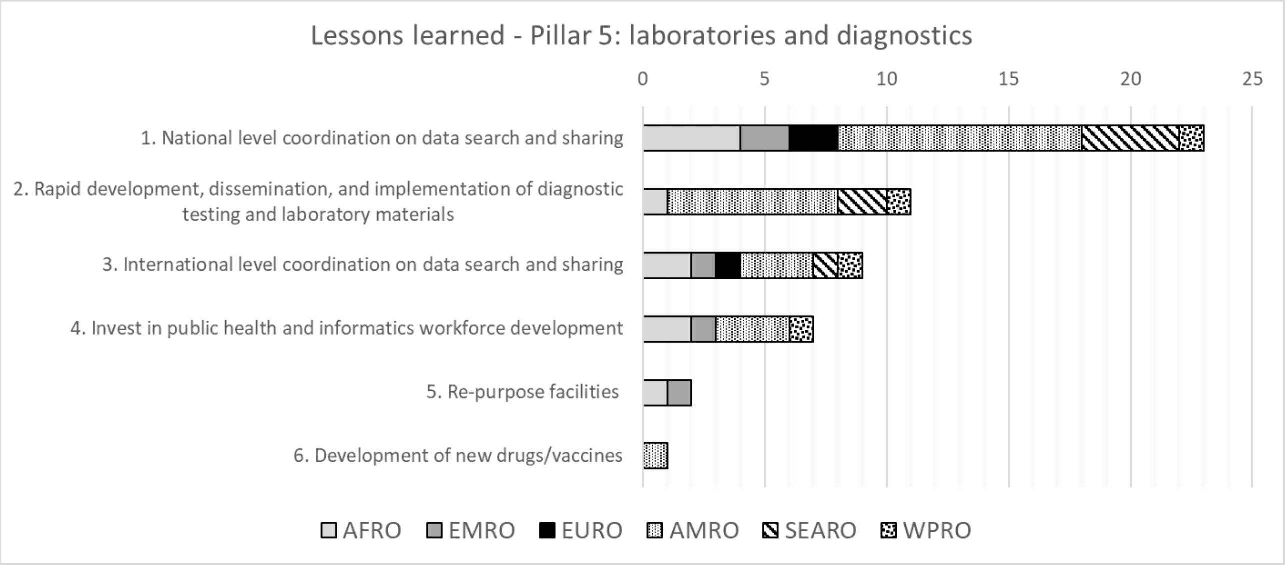

Figure S5. Pillar 5: Ranking of lessons learned and number of their citations in papers from different WHO Regions

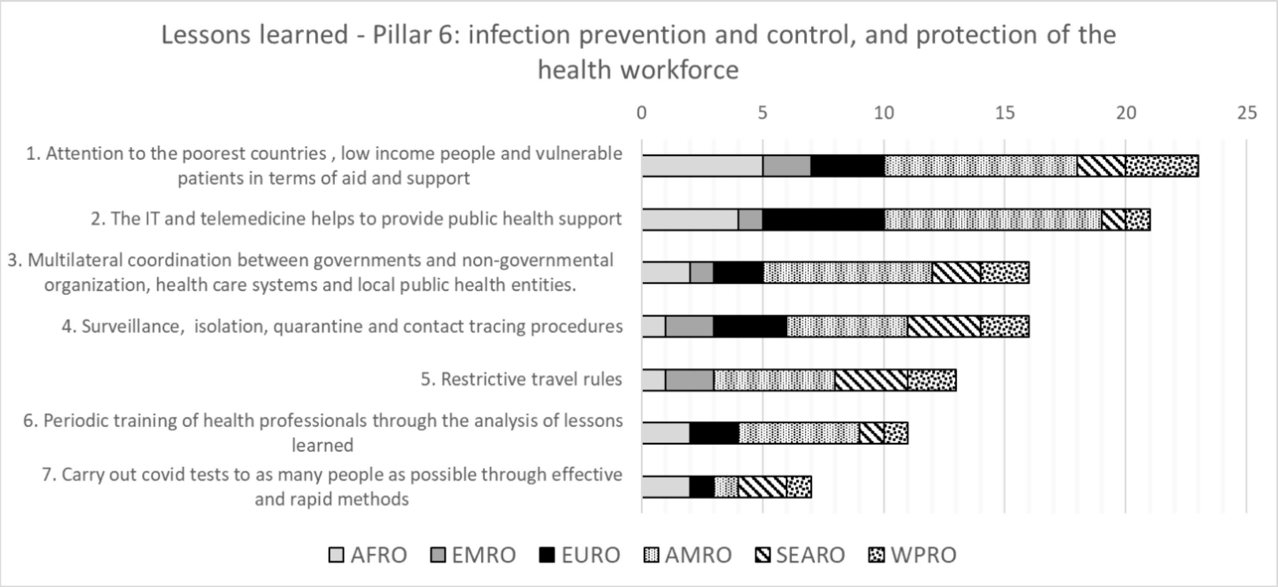

Figure S6. Pillar 6: Ranking of lessons learned and number of their citations in papers from different WHO Regions

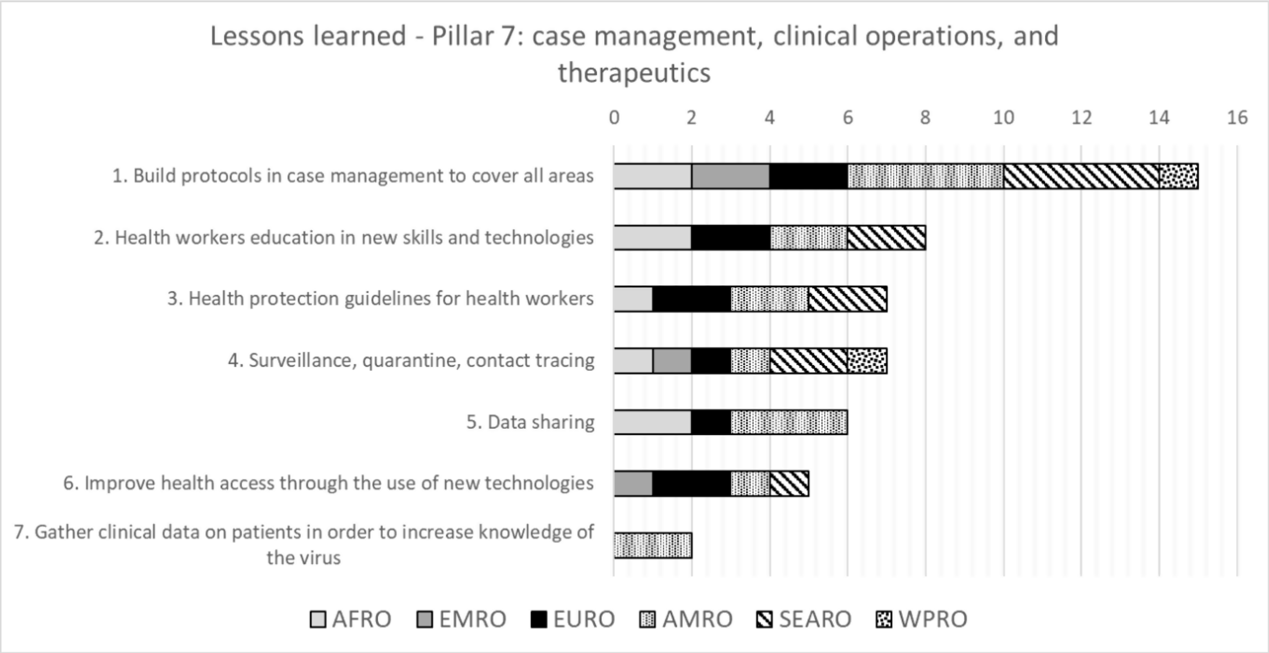

Figure S7. Pillar 7: Ranking of lessons learned and number of their citations in papers from different WHO Regions

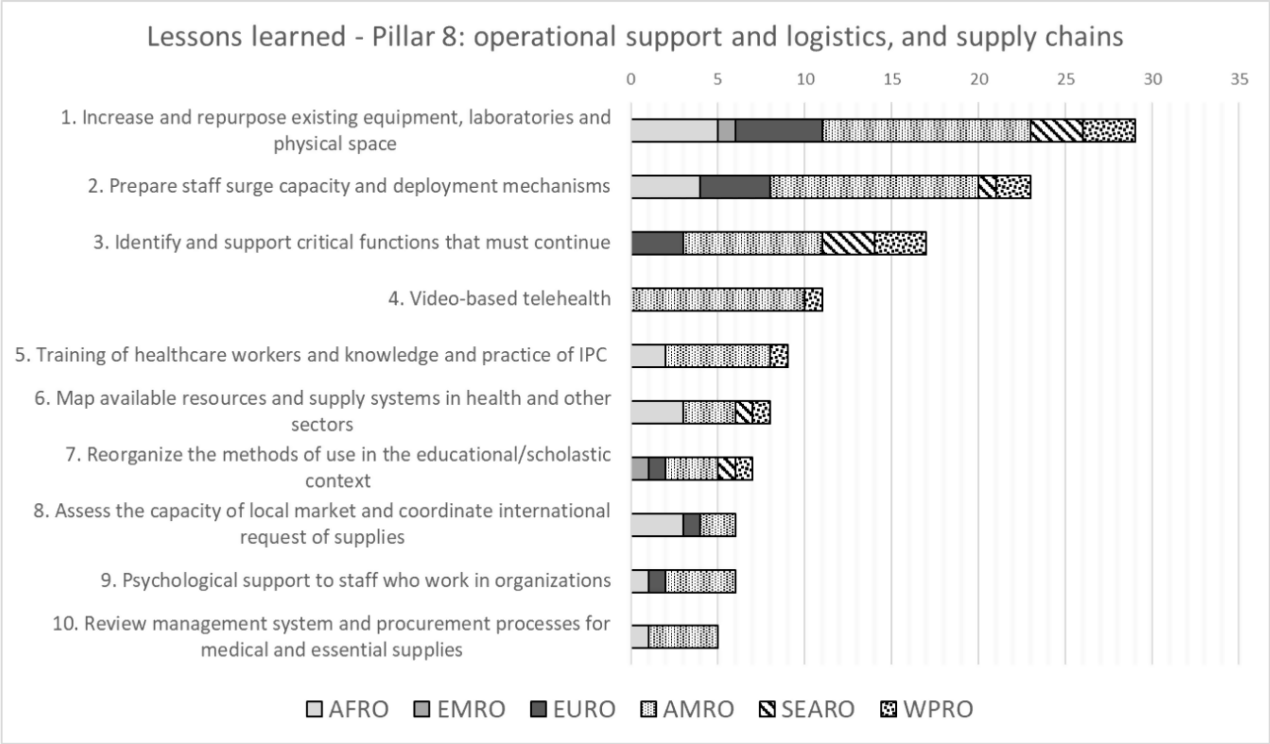

Figure S8. Pillar 8: Ranking of lessons learned and number of their citations in papers from different WHO Regions

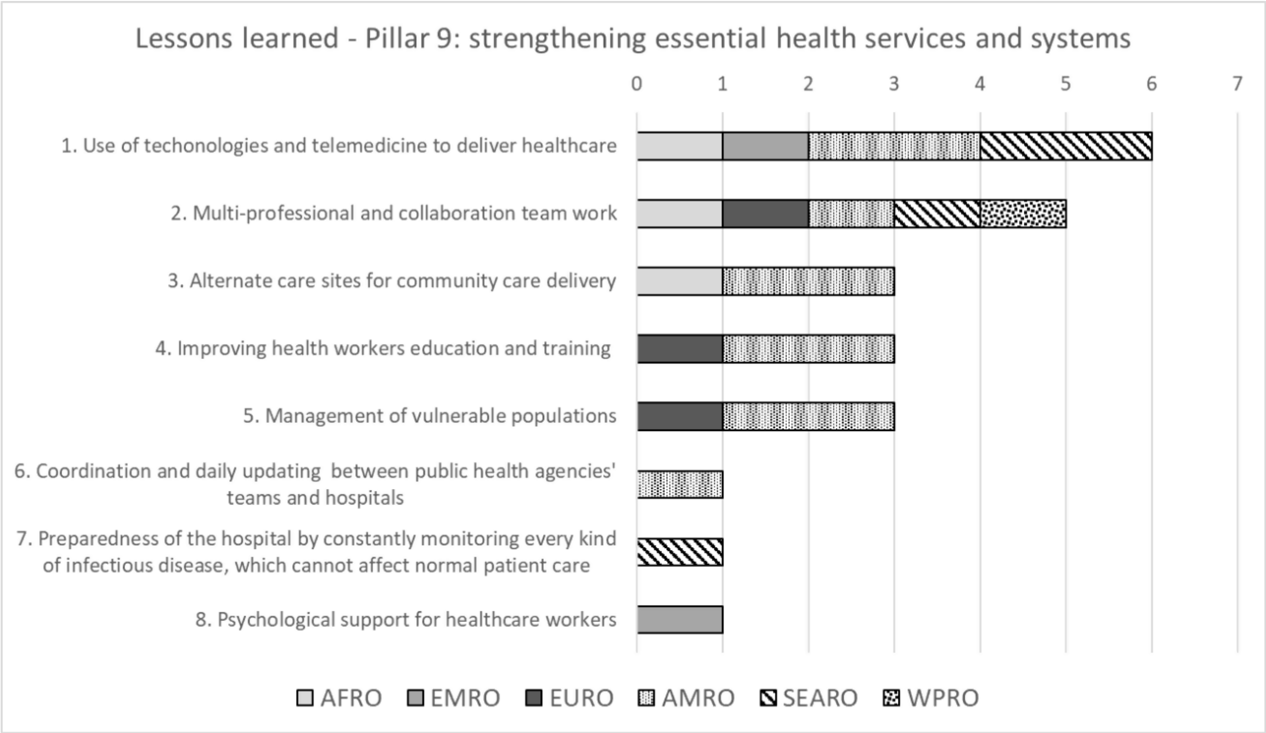

Figure S9. Pillar 9: Ranking of lessons learned and number of their citations in papers from different WHO Regions

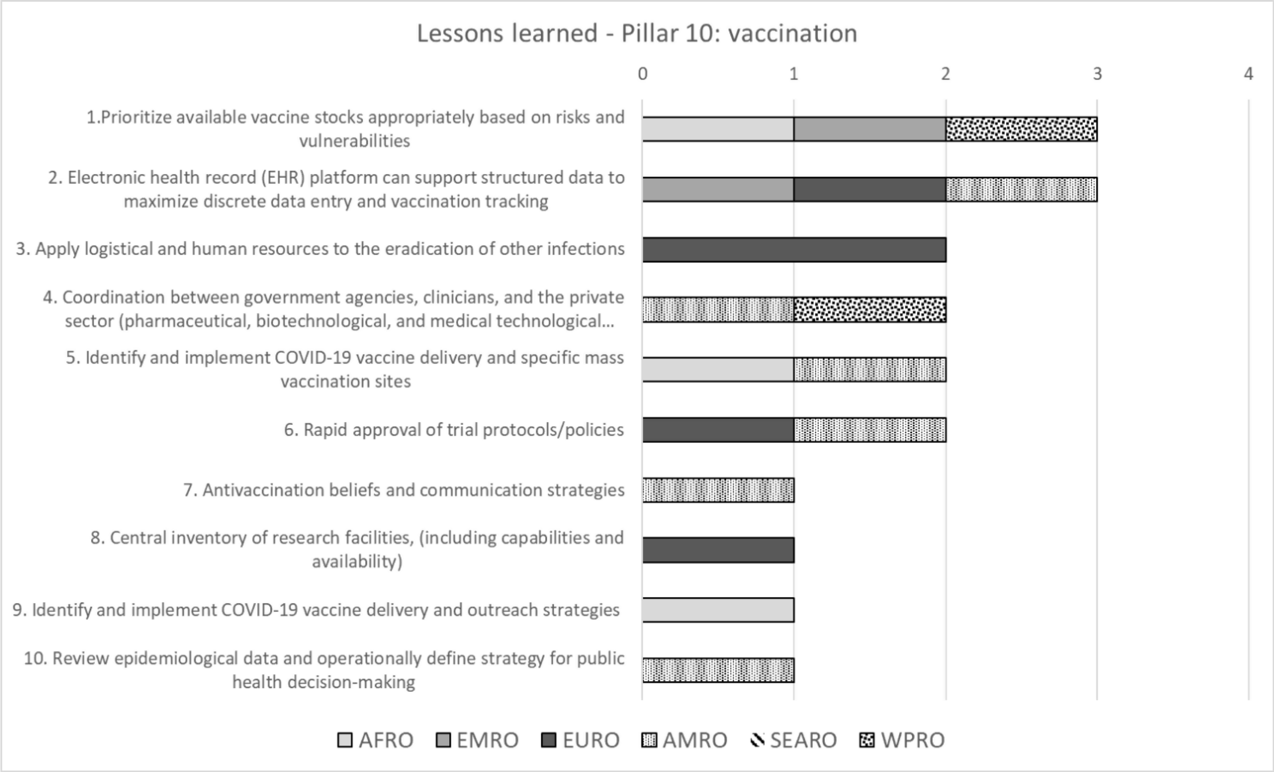

Figure S10. Pillar 10: Ranking of lessons learned and number of their citations in papers from different WHO Regions

Table S1. Studies included and corresponding WHO Pillars public health areas.

Included studies	Pillars
1. Abera A, Belay H, Zewude A, Gidey B, Nega D, Dufera B, et al. Establishment of COVID-19 testing laboratory in resource-limited settings: challenges and prospects reported from Ethiopia. *Glob Health Action*. 2020;13(1):1841963.	1, 5, 8
2. Aborode AT, Hasan MM, Jain S, Okereke M, Adedeji OJ, Karra-Aly A, et al. Impact of poor disease surveillance system on COVID-19 response in africa: Time to rethink and rebuilt. *Clin Epidemiol Glob Health*. 2021;12:100841.	1, 3, 8
3. Abram J, Gasteiger L, Putzer G, Spraider P, Mathis S, Hell T, et al. Impact of COVID-19 Related Lockdown on the Frequency of Acute and Oncological Surgeries-Lessons Learned From an Austrian University Hospital. *J Med Internet Res*. 2020;22(11):e21099.	3, 8
4. Ahmed K, Bukhari MA, Mlanda T, Kimenyi JP, Wallace P, Okot Lukoya C, et al. Novel Approach to Support Rapid Data Collection, Management, and Visualization During the COVID-19 Outbreak Response in the World Health Organization African Region: Development of a Data Summarization and Visualization Tool. *JMIR Public Health Surveill*. 2020;6(4):e20355..	1, 3, 7
5. Al Awaidy ST, Khamis F, Al Attar F, Razzaq NA, Al Dabal L, Al Enani M, et al. COVID-19 in the Gulf Cooperation Council Member States: An Evidence of Effective Response. *Oman Med J*. 2021;36(5):e300.	1, 2, 3, 4, 5, 6, 7, 8, 9
6. Al Ghafri T, Al Ajmi F, Anwar H, Al Balushi L, Al Balushi Z, Al Fahdi F, et al. The Experiences and Perceptions of Health-Care Workers During the COVID-19 Pandemic in Muscat, Oman: A Qualitative Study. *J Prim Care Community Health*. 2020;11:2150132720967514.	1, 7, 8, 9
7. Al Serouri AA, Ghaleb YA, Al Aghbari LA, Al Amad MA, Alkohlani AS, Almoayed KA, et al. Field Epidemiology Training Program Response to COVID-19 During a Conflict: Experience From Yemen. *Front Public Health*. 2021;9:688119.	1, 2, 3, 4, 5, 6, 7, 8
8. Aliyu S, Norful AA, Schroeder K, Odlum M, Glica B, Travers JL. The powder keg: Lessons learned about clinical staff preparedness during the early phase of the COVID-19 pandemic. *Am J Infect Control*. 2021;49(4):478-483.	6, 8
9. Al-Sakkaf E, Ghaleb Y, Al-Dabis E, Qairan M, Al Amad M, Al Serouri A, et al. First COVID-19 cases with high secondary infection among health workers, Sana'a capital, April 2020: Lessons learned and future opportunities. *Int J Infect Dis*. 2021;110 Suppl 1:S6-S10.	1, 7
10. Arafa M, Nesar S, Abu-Jabeh H, Jayme MOR, Kalairajah Y. COVID-19 pandemic and hip	6, 7, 8, 9

fractures: impact and lessons learned. *Bone Jt Open*. 2020;1(9):530-540.	
11. Aruru M, Truong HA, Clark S. Pharmacy Emergency Preparedness and Response (PEPR): a proposed framework for expanding pharmacy professionals' roles and contributions to emergency preparedness and response during the COVID-19 pandemic and beyond. *Res Social Adm Pharm*. 2021;17(1):1967-1977.	2, 6
12. Ashinyo ME. Ghana beyond the epi-curve: initial lessons learned from the implementation of infection prevention and control measures in the COVID-19 response. *Pan Afr Med J*. 2021;38:18.	1, 2, 6, 8
13. Asirvatham ES, Lakshmanan J, Sarman CJ, Joy M. Demystifying the varying case fatality rates (CFR) of COVID-19 in India: Lessons learned and future directions. *J Infect Dev Ctries*. 2020;14(10):1128-1135.	1, 3, 6, 8
14. Assefa N, Hassen JY, Admassu D, Brhane M, Deressa M, Marami D, et al. COVID-19 Testing Experience in a Resource-Limited Setting: The Use of Existing Facilities in Public Health Emergency Management. *Front Public Health*. 2021;9:675553.	1, 3, 5, 6, 8
15. Assiri A, Al-Tawfiq JA, Alkhalifa M, Al Duhailan H, Al Qahtani S, Dawas RA, et al. Launching COVID-19 vaccination in Saudi Arabia: Lessons learned, and the way forward. *Travel Med Infect Dis*. 2021;43:102119.	1, 6, 8, 10
16. B Harpin S. Creating COVID-19 alternate care site trainings for interprofessional teams. *Public Health Nurs*. 2020;37(6):941-945.	7, 8, 9
17. Bacchus P, Nissen K, Berg J, Bråve A, Gyll J, Larsson C, et al. Civil-Military Collaboration to Facilitate Rapid Deployment of a Mobile Laboratory in Early Response to COVID-19: A High-Readiness Exercise. *Health Secur*. 2021;19(5):488-497.	5, 9
18. Barnert E, Kwan A, Williams B. Ten Urgent Priorities Based on Lessons Learned From More Than a Half Million Known COVID-19 Cases in US Prisons. *Am J Public Health*. 2021;111(6):1099-1105.	6, 8
19. Basit MA, Lehmann CU, Medford RJ. Managing Pandemics with Health Informatics: Successes and Challenges. *Yearb Med Inform*. 2021;30(1):17-25.	1
20. Bookman RJ, Cimino JJ, Harle CA, Kost RG, Mooney S, Pfaff E, et al. Research informatics and the COVID-19 pandemic: Challenges, innovations, lessons learned, and recommendations. *J Clin Transl Sci*. 2021;5(1):e110.	2, 5, 7, 8, 9
21. Bowsher G, Bernard R, Sullivan R. A Health Intelligence Framework for Pandemic Response: Lessons from the UK Experience of COVID-19. *Health Secur*. 2020;18(6):435-443.	1, 2, 8
22. Brenner AB, Knaub M, Robinson K, Lotspeich M, Eisen J. Building Resilience in the Face of Crisis:	2, 6

Lessons Learned from a Community Behavioral Healthcare Organization. *J Behav Health Serv Res*. 2022;49(3):406-413.	
23. Bright D, Brown G, Roberts RJ, Cottrell S, Gould A, Jesurasa A, et al. COVID-19 contact tracing: The Welsh experience. *Public Health Pract (Oxf)*. 2020;1:100035.	1, 3, 4, 6, 7, 8
24. Broach JP, Lowell M, Brown O, Martin C, Muller M, Shirshac J, et al. A Citywide Approach to SARS-CoV2 Testing. *Front Public Health*. 2021;9:695442.	1, 3
25. Broadway KM, Schwartz-Watjen KT, Swiatecka AL, Hadeed SJ, Owens AN, Batni SR, et al. Operational Considerations in Global Health Modeling. *Pathogens*. 2021;10(10):1348.	1, 2, 3, 5, 6
26. Buzelli ML, Boyce T. The Privatization of the Italian National Health System and its Impact on Health Emergency Preparedness and Response: The COVID-19 Case. *Int J Health Serv*. 2021;51(4):501-508.	1, 8
27. Callaway D, Runge J, Mullen L, Rentz L, Staley K, Stanford M, et al. Risk and the Republican National Convention: Application of the Novel COVID-19 Operational Risk Assessment. *Disaster Med Public Health Prep*. 2021;1-6.	1
28. Castillo J, Fremion E, Morrison-Jacobus M, Bolin R, Perez A, Acosta E, et al. Think globally, act locally: Quality improvement as a catalyst for COVID-19 related care during the transitional years. *J Pediatr Rehabil Med*. 2021;14(4):691-697.	6, 10
29. Chen Z, Cao C, Yang G. Coordinated multi-sectoral efforts needed to address the COVID-19 pandemic: lessons from China and the United States. *Glob Health Res Policy*. 2020;5:22.	1, 3, 6, 8
30. Chidavaenzi NZ, Agathis N, Lees Y, Stevens H, Clark J, Reede D, Kunkel A, Balajee SA. Implementation of a COVID-19 Screening Testing Program in a Rural, Tribal Nation: Experience of the San Carlos Apache Tribe, January-February 2021. *Public Health Rep*. 2022;137(2):220-225.	1, 3
31. Chimene M, Nhari LG, Dzobo M, Mhango M, Dzinamarira T. COVID-19 rapid response in a limited resource setting (notes from the field): Chinhoyi Provincial Hospital, Mashonaland West Province, Zimbabwe. *Pan Afr Med J*. 2021;39:111.	1, 2, 3, 7, 9
32. Coccolini F, Cicuttin E, Cremonini C, Tartaglia D, Viaggi B, Kuriyama A, et al. A pandemic recap: lessons we have learned. *World J Emerg Surg*. 2021;16(1):46	1, 2, 7, 8, 9, 10
33. Coumare VN, Pawar SJ, Manoharan PS, Pajanivel R, Shanmugam L, Kumar H, et al. COVID-19 Pandemic-Frontline Experiences and Lessons Learned From a Tertiary Care Teaching Hospital at a Suburban Location of Southeastern India. *Front Public Health*. 2021;9:673536.	2, 3, 4, 7, 9

34. Dariotis JK, Sloane SM, Smith RL. "I took it off most of the time 'cause I felt comfortable": unmasking, trusted others, and lessons learned from a coronavirus disease 2019 reinfection: a case report. *J Med Case Rep*. 2021;15(1):557.	2, 6
35. Denis JL, Potvin L, Rochon J, Fournier P, Gauvin L. On redesigning public health in Québec: lessons learned from the pandemic. *Can J Public Health*. 2020;111(6):912-920.	1, 2, 3
36. Doraiswamy S, Mamtani R, Cheema S. An in-depth analysis of 10 epidemiological terminologies used in the context of COVID-19. *Scand J Public Health*. 2021;14034948211057736.	2
37. Doumbia S, Sow Y, Diakite M, Lau CY. Coordinating the research response to COVID-19: Mali's approach. *Health Res Policy Syst*. 2020;18(1):105.	1, 2, 3, 6, 8
38. Dumproff JB, Bishara J, Copeland N, Fredricks T. Integrating US National Guard with Public Health Partners at COVID-19 Testing Sites in West Virginia Counties with High Rural and Minority Populations: Lessons Learned. *Health Secur*. 2022;20(1):58-64.	2, 6
39. Duong DM, Le VT, Ha BTT. Controlling the COVID-19 Pandemic in Vietnam: Lessons From a Limited Resource Country. *Asia Pac J Public Health*. 2020;32(4):161-162.	1, 2, 3, 4, 6, 7
40. Dys S, Winfree J, Carder P, Zimmerman S, Thomas KS. Coronavirus Disease 2019 Regulatory Response in United States-Assisted Living Communities: Lessons Learned. *Front Public Health*. 2021;9:661042.	1
41. Edge HM, Carlucci S, Lu D. The role of Force Health Protection in the Canadian Armed Forces' response to the COVID-19 pandemic. *Can Commun Dis Rep*. 2020;46(9):279-281.	1, 8
42. El-Jardali F, Bou-Karroum L, Fadlallah R. Amplifying the role of knowledge translation platforms in the COVID-19 pandemic response. *Health Res Policy Syst*. 2020;18(1):58.	1, 2, 5
43. Elman A, Breckman R, Clark S, Gottesman E, Rachmuth L, Reiff M, et al. Effects of the COVID-19 Outbreak on Elder Mistreatment and Response in New York City: Initial Lessons. J Appl Gerontol. 2020 Jul;39(7):690-699. doi: 10.1177/0733464820924853. *J Appl Gerontol*. 2020;39(7):690-699.	1
44. Endres-Dighe S, Jones K, Hadley E, Preiss A, Kery C, Stoner M, et al. Lessons learned from the rapid development of a statewide simulation model for predicting COVID-19's impact on healthcare resources and capacity. *PLoS One*. 2021;16(11):e0260310.	1, 2, 3, 6
45. Fader KA, Zhang J, Menetski JP, Thadhani RI, Antman EM, Friedman GS, et al. A Biomarker-	5, 10

Centric Approach to Drug Discovery and Development: Lessons Learned from the Coronavirus Disease 2019 Pandemic. *J Pharmacol Exp Ther*. 2021;376(1):12-20.	
46. Flynn EF, Kuhn E, Shaik M, Tarr E, Scattolini N, Ballantine A. Drive-Through COVID-19 Testing During the 2020 Pandemic: A Safe, Efficient, and Scalable Model for Pediatric Patients and Health Care Workers. *Acad Pediatr*. 2020;20(6):753-755.	3, 6
47. Foraker RE, Lai AM, Kannampallil TG, Woeltje KF, Trolard AM, Payne PRO. Transmission dynamics: Data sharing in the COVID-19 era. *Learn Health Syst*. 2020;5(1):e10235.	1, 3, 6
48. Gibson L, Fahey N, Hafer N, Buchholz B, Dunlap D, Murphy R, et al. The RADx Tech Clinical Studies Core: A Model for Academic Based Clinical Studies. *IEEE Open J Eng Med Biol*. 2021;2:152-157.	2, 8
49. Goldberg SA, Callaway D, Resnick-Ault D, Mandavia S, Martinez R, Bass M, et al. Critical Concepts for COVID-19 Mass Vaccination Site Operations. *Disaster Med Public Health Prep*. 2021;1-7.	5, 6, 8, 10
50. Gravelin M, Wright J, Holbein MEB, Berro M, Brown JS, Mashour GA, et al. Role of CTSA institutes and academic medical centers in facilitating preapproval access to investigational agents and devices during the COVID-19 pandemic. *J Clin Transl Sci*. 2021;5(1):e94.	1
51. Gur-Arie R, Johnson S, Collins M. Advancing child health and educational equity during the COVID-19 pandemic through science and advocacy. *Isr J Health Policy Res*. 2022;11(1):3.	1, 5, 8
52. Ha KM. Changes in awareness on face mask use in Korea. *Public Health Nurs*. 2022;39(2):506-508.	6
53. Hanna J, Chen T, Portales-Castillo C, Said M, Bulnes R, Newhart D, et al. THE VALUE OF A REGIONAL 'LIVING' COVID-19 REGISTRY AND THE CHALLENGES OF KEEPING IT ALIVE. *Perspect Health Inf Manag*. 2021;18(3):1d.	3
54. Hansen T, Sevenius Nilsen T, Knapstad M, Skirbekk V, Skogen J, Vedaa Ø, et al. Covid-fatigued? A longitudinal study of Norwegian older adults' psychosocial well-being before and during early and later stages of the COVID-19 pandemic. *Eur J Ageing*. 2021;1-11.	1
55. Hettiarachchi D, Noordeen N, Gamakaranage C, Somarathne EARBD, Jayasinghe S. Ethical Responses to the COVID-19 Pandemic-Lessons from Sri Lanka. *Asian Bioeth Rev*. 2020;13(2):225-233.	1, 2
56. Heudorf U, Müller M, Schmehl C, Gasteyer S, Steul K. COVID-19 in long-term care facilities in Frankfurt am Main, Germany: incidence, case	1

reports, and lessons learned. *GMS Hyg Infect Control*. 2020;15:Doc26.	
57. Holden B, Quinney A, Padfield S, Morton W, Coles S, Manley P, et al. COVID-19: public health management of the first two confirmed cases identified in the UK. *Epidemiol Infect*. 2020;148:e194.	7
58. Hunger J, Schumann H. How to achieve quality assurance, shared ethics and efficient teambuilding? Lessons learned from interprofessional collaboration during the COVID-19 pandemic. *GMS J Med Educ*. 2020;37(7):Doc79.	8
59. Impouma B, Wolfe CM, Mboussou F, Farham B, Saturday T, Pervilhac C, et al. Monitoring and evaluation of COVID-19 response in the WHO African region: challenges and lessons learned. *Epidemiol Infect*. 2021;149:e98	1, 3, 4, 5, 8
60. Jungo S, Moreau N, Mazevet ME, Ejeil AL, Biosse Duplan M, Salmon B,et al. Prevalence and risk indicators of first-wave COVID-19 among oral health-care workers: A French epidemiological survey. *PLoS One*. 2021;16(2):e0246586.	6
61. Kadakia KT, Howell MD, DeSalvo KB. Modernizing Public Health Data Systems: Lessons From the Health Information Technology for Economic and Clinical Health (HITECH) Act. *JAMA*. 2021;326(5):385-386.	1, 3, 5
62. Kalyanaraman N, Fraser MR. Containing COVID-19 Through Contact Tracing : A Local Health Agency Approach. *Public Health Rep*. 2021;136(1):32-38.	3
63. Kroll KH, Larsen S, Lamb K, Davies WH, Cipriano D, deRoon-Cassini TA, et al. Responding to the Psychological Needs of Health-Care Workers During the COVID-19 Pandemic: Case Study from the Medical College of Wisconsin. *J Clin Psychol Med Settings*. 2022;29(1):150-161.	8
64. Krouss M, Allison MG, Rios S, Bringardner BD, Langston MD, Sokol SI, et al. Rapid Implementation of Telecritical Care Support During a Pandemic: Lessons Learned During the Coronavirus Disease 2020 Surge in New York City. *Crit Care Explor*. 2020;2(11):e0271.	8
65. Kuriakose S, Singh K, Pau AK, Daar E, Gandhi R, Tebas P, et al. Developing Treatment Guidelines During a Pandemic Health Crisis: Lessons Learned From COVID-19. *Ann Intern Med*. 2021;174(8):1151-1158.	1, 2, 6, 7, 8
66. Lal S, Gleeson J, Rivard L, D'Alfonso S, Joober R, Malla A, et al. Adaptation of a Digital Health Innovation to Prevent Relapse and Support Recovery in Youth Receiving Services for First-Episode Psychosis: Results From the Horyzons-Canada Phase 1 Study. *JMIR Form Res*. 2020;4(10):e19887.	1

67. Lami F, Rashak HA, Khaleel HA, Mahdi SG, Adnan F, Khader YS, et al. Iraq experience in handling the COVID-19 pandemic: implications of public health challenges and lessons learned for future epidemic preparedness planning. *J Public Health (Oxf)*. 2021;43(Suppl 3):iii19-iii28.	1, 3, 4, 6
68. Lee SM, Lee D. Lessons Learned from Battling COVID-19: The Korean Experience. *Int J Environ Res Public Health*. 2020;17(20):7548.	1, 2, 3, 6, 8
69. Leite JA, Gresh L, Vicari A, Gabastou JM, Perez E, Aldighieri S; et al. COVID-19 laboratory preparedness and response in the Americas Region: Lessons learned. *PLoS One*. 2021;16(6):e0253334.	1, 3, 5, 8
70. Leser KA, Hay MC, Henebry B, Virden J, Patel M, Luttrell-Freeman J, et al. An Academic-Health Department Community Partnership to Expand Disease Investigation and Contact Tracing Capacity and Efficiency During the COVID-19 Pandemic. *J Public Health Manag Pract*. 2022;28(1):E16-E22.	3
71. Levin-Zamir D, Sorensen K, Su TT, Sentell T, Rowlands G, Messer M, et al. Health promotion preparedness for health crises - a 'must' or 'nice to have'? Case studies and global lessons learned from the COVID-19 pandemic. *Glob Health Promot*. 2021;28(2):27-37.	1, 2, 8
72. Marcos-Garcia P, Carmona-Moreno C, López-Puga J, Ruiz-Ruano García AM. COVID-19 pandemic in Africa: Is it time for water, sanitation and hygiene to climb up the ladder of global priorities? *Sci Total Environ*. 2021;791:148252.	1, 6, 8
73. Margusino-Framiñán L, Illarro-Uranga A, Lorenzo-Lorenzo K, Monte-Boquet E, Márquez-Saavedra E, Fernández-Bargiela N, et al. Pharmaceutical care to hospital outpatients during the COVID-19 pandemic. Telepharmacy. *Farm Hosp*. 2020;44(7):61-65. Published 2020 Jun 13.	6
74. Marsh EE, Kappelman MD, Kost RG, Mudd-Martin G, Shannon J, Stark LA, et al. Community engagement during COVID: A field report from seven CTSAs. *J Clin Transl Sci*. 2021;5(1):e104.	1, 2
75. McLaughlin HP, Hiatt BC, Russell D, Carlson CM, Jacobs JR, Perez-Osorio AC, et al. COVID-19 Response Efforts of Washington State Public Health Laboratory: Lessons Learned. *Am J Public Health*. 2021;111(5):867-875.	1
76. Medley AM, Marston BJ, Toda M, Kobayashi M, Weinberg M, Moriarty LF, et al. Use of US Public Health Travel Restrictions during COVID-19 Outbreak on Diamond Princess Ship, Japan, February-April 2020. *Emerg Infect Dis*. 2021;27(3):710-718.	4, 6
77. Mellins CA, Mayer LES, Glasofer DR, Devlin MJ, Albano AM, Nash SS, et al. Supporting the well-being of health care providers during the COVID-19	8

pandemic: The CopeColumbia response. *Gen Hosp Psychiatry*. 2020;67:62-69.	
78. Melvin CL, Sterba KR, Gimbel R, Lenert LA, Cartmell KB; SC Safer Together Team. Dissemination and Implementation of a Google Apple Exposure Notification System for COVID-19 Risk Mitigation at a National Public University: Protocol for a Pilot Evaluation Study in a Real-World Setting. *JMIR Res Protoc*. 2022;11(1):e32567.	6
79. Merchant T, Hormozian S, Smith RS, Pendergrast T, Siddiqui A, Wen Z, et al. Ethical Principles in Personal Protective Equipment Inventory Management Decisions and Partnerships Across State Lines During the COVID-19 Pandemic. *Public Health Rep*. 2022;137(2):208-212.	6, 8
80. Messinger M, McNeill MM. Community Hospital Perioperative Services Department Responds to the COVID-19 Pandemic. *AORN J*. 2021;113(2):165-178.	8
81. Michener L, Aguilar-Gaxiola S, Alberti PM, Castaneda MJ, Castrucci BC, Harrison LM, et al. Engaging With Communities - Lessons (Re)Learned From COVID-19. *Prev Chronic Dis*. 2020;17:E65.	1, 2, 8
82. Mitchell SH, Bulger EM, Duber HC, Greninger AL, Ong TD, Morris SC, et al. Western WA COVID-19 Expert Panel. Western Washington State COVID-19 Experience: Keys to Flattening the Curve and Effective Health System Response. *J Am Coll Surg*. 2020;231(3):316-324.e1.	1, 3, 6, 7, 8, 9
83. Moore SL, Portz JD, Santodomingo M, Elsbernd K, McHale M, Massone J. Using Telehealth for Hospice Reauthorization Visits: Results of a Quality Improvement Analysis. *J Pain Symptom Manage*. 2020;60(3):e22-e27.	8
84. Moran JH, Kessler L, Moylan J, Forrest C, Boehme K, Kennedy J, et al. Modifying laboratory testing via home brew during the COVID-19 pandemic. *J Clin Transl Sci*. 2021;5(1):e93.	5
85. Morozova O, Li ZR, Crawford FW. One year of modeling and forecasting COVID-19 transmission to support policymakers in Connecticut. *Sci Rep*. 2021;11(1):20271.	1
86. Mueller UE, Omosehin O, Akinkunmi AE, Ayanbadejo JO, Somefun EO, Momah-Haruna AP. Contact Tracing in an African Megacity during COVID 19: Lessons Learned. *Afr J Reprod Health*. 2020;24(s1):27-31.	6
87. Nachega JB, Atteh R, Ihekweazu C, Sam-Agudu NA, Adejumo P, Nsanzimana S, et al. Contact Tracing and the COVID-19 Response in Africa: Best Practices, Key Challenges, and Lessons Learned from Nigeria, Rwanda, South Africa, and Uganda. *Am J Trop Med Hyg*. 2021;104(4):1179-1187.	3, 6

88. Nam NH, Quy PN, Pham TM, Branch J. No new community COVID-19 infection in four consecutive weeks: what lesson can be learned from Vietnam. *J Infect Dev Ctries*. 2020;14(10):1125-1127.	1, 3, 4, 8
89. Negro-Calduch E, Azzopardi-Muscat N, Nitzan D, Pebody R, Jorgensen P, Novillo-Ortiz D. Health Information Systems in the COVID-19 Pandemic: A Short Survey of Experiences and Lessons Learned From the European Region. *Front Public Health*. 2021;9:676838.	1, 2, 3, 8
90. Neil-Sztramko SE, Belita E, Traynor RL, Clark E, Hagerman L, Dobbins M. Methods to support evidence-informed decision-making in the midst of COVID-19: creation and evolution of a rapid review service from the National Collaborating Centre for Methods and Tools. *BMC Med Res Methodol*. 2021;21(1):231.	1, 5
91. Neogi SB, Preetha GS. Assessing health systems' responsiveness in tackling COVID-19 pandemic. *Indian J Public Health*. 2020;64(Supplement):S211-S216.	1, 3, 5, 8
92. Nguyen AL, Brown B, Tantawi ME, Ndembi N, Okeibunor J, Mohammed A, et al. Time to Scale-up Research Collaborations to Address the Global Impact of COVID-19 - A Commentary. *Health Behav Policy Rev*. 2021;8(3):277-280.	1
93. Noce E, Zorzanello M, Patel D, Kodali R. Management of COVID-19 in an Outpatient Dialysis Program. *Nephrol Nurs J*. 2020;47(5):423-427.	6
94. Obaseki DE, Osaigbovo II, Ogboghodo EO, Adeleye O, Akoria OA, Oko-Oboh GA, et al. Preparedness and response of a tertiary hospital to the COVID-19 pandemic in Nigeria: challenges, opportunities and lessons. *Trans R Soc Trop Med Hyg*. 2021;115(7):727-730.	6, 8
95. Paltiel O, Hochner H, Chinitz D, Clarfield AM, Gileles-Hillel A, Lahad A, et al. Academic activism on behalf of children during the COVID-19 pandemic in Israel; beyond public health advocacy. *Isr J Health Policy Res*. 2021;10(1):48.	1, 2, 6
96. Papadimos TJ, Soghoian SE, Nanayakkara P, Singh S, Miller AC, Saddikuti V, et al. COVID-19 Blind Spots: A Consensus Statement on the Importance of Competent Political Leadership and the Need for Public Health Cognizance. *J Glob Infect Dis*. 2020;12(4):167-190.	1, 2, 8
97. Patel A. Preventing COVID-19 Amid Public Health and Urban Planning Failures in Slums of Indian Cities. *World Med Health Policy*. 2020;12(3):266-273.	1, 6, 7
98. Peng W, Berry EM. Coping with the Challenges of COVID-19 Using the Sociotype Framework: A Rehearsal for the Next Pandemic. *Rambam Maimonides Med J*. 2021;12(1):e0005.	1, 2, 4, 6, 8

99. Quah LJJ, Tan BKK, Fua TP, Wee CPJ, Lim CS, Nadarajan G, et al. Reorganising the emergency department to manage the COVID-19 outbreak. *Int J Emerg Med*. 2020;13(1):32.	7, 8, 9
100. Ren J, Yang X, Xu Z, Lei W, Yang K, Kong Y, et al. Prevention of nosocomial COVID-19 infections in otorhinolaryngology-head and neck surgery. *World J Otorhinolaryngol Head Neck Surg*. 2020;6(Suppl 1):S6-S10.	6
101. Resnicow K, Bacon E, Yang P, Hawley S, Van Horn ML, An L. Novel Predictors of COVID-19 Protective Behaviors Among US Adults: Cross-sectional Survey. *J Med Internet Res*. 2021;23(4):e23488.	2, 10
102. Rick F, Odoke W, van den Hombergh J, Benzaken AS, Avelino-Silva VI. Impact of coronavirus disease (COVID-19) on HIV testing and care provision across four continents. *HIV Med*. 2022;23(2):169-177.	8
103. Rodríguez DC, Jessani NS, Zunt J, Ardila-Gómez S, Muwanguzi PA, Atanga SN, et al. Experiential Learning and Mentorship in Global Health Leadership Programs: Capturing Lessons from Across the Globe. *Ann Glob Health*. 2021;87(1):61.	1
104. Rodriguez NM, Lahey AM, MacNeill JJ, Martinez RG, Teo NE, Ruiz Y. Homelessness during COVID-19: challenges, responses, and lessons learned from homeless service providers in Tippecanoe County, Indiana. *BMC Public Health*. 2021;21(1):1657.	1, 2, 7, 8, 9
105. Ros F, Kush R, Friedman C, Gil Zorzo E, Rivero Corte P, Rubin JC, et al. Addressing the Covid-19 pandemic and future public health challenges through global collaboration and a data-driven systems approach. *Learn Health Syst*. 2020;5(1):e10253.	1, 5
106. Ruebush E, Fraser MR, Poulin A, Allen M, Lane JT, Blumenstock JS. COVID-19 Case Investigation and Contact Tracing: Early Lessons Learned and Future Opportunities. *J Public Health Manag Pract*. 2021;27 Suppl 1, COVID-19 and Public Health: Looking Back, Moving Forward:S87-S97.	1, 6
107. Ryan BJ, Muehlenbein MP, Allen J, Been J, Boyd K, Brickhouse M, et al. Sustaining University Operations During the COVID-19 Pandemic. *Disaster Med Public Health Prep*. 2021;1-9.	3, 5, 6
108. Salvatore AL, Ortiz J, Booker E; LPC;3, Katurakes N, Moore CC, Johnson CPA, et al. Engaging Community Health Workers and Social Care Staff as Social First Responders during the COVID-19 Crisis. *Dela J Public Health*. 2020;6(2):92-95.	2
109. Sauer MA, Truelove S, Gerste AK, Limaye RJ. A Failure to Communicate? How Public Messaging Has Strained the COVID-19 Response in the United States. *Health Secur*. 2021;19(1):65-74.	2

110. Schuftan C. Vietnam's Containment of COVID-19: Why the Coronavirus Mortality has Been So Low. *Int J Health Serv*. 2021;51(2):238-241.	1, 3, 4, 5, 6, 7
111. Shah A, Guessi M, Wali S, Ware P, McDonald M, O'Sullivan M, et al. The Resilience of Cardiac Care Through Virtualized Services During the COVID-19 Pandemic: Case Study of a Heart Function Clinic. *JMIR Cardio*. 2021;5(1):e25277.	1, 8
112. Shelby T, Schenck C, Weeks B, Goodwin J, Hennein R, Zhou X, et al. Lessons Learned From COVID-19 Contact Tracing During a Public Health Emergency: A Prospective Implementation Study. *Front Public Health*. 2021;9:721952.	3, 6
113. Shimizu K, Negita M. Lessons Learned from Japan's Response to the First Wave of COVID-19: A Content Analysis. *Healthcare (Basel)*. 2020;8(4):426.	1, 2, 3, 4, 6, 7, 8
114. Sklar DP. COVID-19: Lessons From the Disaster That Can Improve Health Professions Education. *Acad Med*. 2020;95(11):1631-1633.	9
115. Sommariva S, Mote J, Ballester Bon H, Razafindraibe H, Ratovozanany D, Rasoamanana V, et al. Social Listening in Eastern and Southern Africa, a UNICEF Risk Communication and Community Engagement Strategy to Address the COVID-19 Infodemic. *Health Secur*. 2021;19(1):57-64.	2
116. Stawicki SP, Jeanmonod R, Miller AC, Paladino L, Gaieski DF, Yaffee AQ, et al. The 2019-2020 Novel Coronavirus (Severe Acute Respiratory Syndrome Coronavirus 2) Pandemic: A Joint American College of Academic International Medicine-World Academic Council of Emergency Medicine Multidisciplinary COVID-19 Working Group Consensus Paper. *J Glob Infect Dis*. 2020;12(2):47-93.	1, 3, 5, 6, 7, 8
117. Stewart T, Day SW, Russell J, Wilbanks C, Likes W, Webb S, et al. Development of a COVID-19 alternate care site from ground zero: A nursing perspective. *Public Health Nurs*. 2020;37(6):889-894.	7, 8
118. Su L, Hong N, Zhou X, He J, Ma Y, Jiang H, et al. Evaluation of the Secondary Transmission Pattern and Epidemic Prediction of COVID-19 in the Four Metropolitan Areas of China. *Front Med (Lausanne)*. 2020;7:171.	1, 3, 6
119. Sudat SEK, Robinson SC, Mudiganti S, Mani A, Pressman AR. Mind the clinical-analytic gap: Electronic health records and COVID-19 pandemic response. *J Biomed Inform*. 2021;116:103715.	1, 3, 5, 7, 9
120. Sullivan ADW, Forehand R, Acosta J, Parent J, Comer JS, Loiselle R, Jones DJ. COVID-19 and the Acceleration of Behavioral Parent Training Telehealth: Current Status and Future Directions. *Cogn Behav Pract*. 2021;28(4):618-629.	8

121. Taher N, Huda MS, Chowdhury TA. COVID-19 and diabetes: What have we learned so far? *Clin Med (Lond)*. 2020;20(4):e87-e90.	2, 8
122. Toney D, Pentella M, Blank E, Becker S. Creating a Blueprint for the Future: Lessons Learned From Public Health Laboratories in the COVID-19 Response. *J Public Health Manag Pract*. 2021;27 Suppl 1, COVID-19 and Public Health: Looking Back, Moving Forward:S101-S105.	5, 8
123. Török ME, Underwood BR, Toshner M, Waddington C, Sidhom E, Sharrocks K, et al. Challenges and opportunities for conducting a vaccine trial during the COVID-19 pandemic in the United Kingdom. *Clin Trials*. 2021;18(5):615-621.	1, 2, 6, 10
124. Tracie EisBrenner1*, Tipples G, Kuschak T, Gilmour M. Laboratory response checklist for infectious disease outbreaks-preparedness and response considerations for emerging threats. *Can Commun Dis Rep*. 2020;46(10):311-321.	5
125. Tran DN, Were PM, Kangogo K, Amisi JA, Manji I, Pastakia SD, et al. Supply-chain strategies for essential medicines in rural western Kenya during COVID-19. *Bull World Health Organ*. 2021;99(5):388-392.	8
126. Tsai JM, Tolan NV, Petrides AK, Kanjilal S, Brigl M, Lindeman NI, et al. How SARS-CoV-2 Transformed the Clinical Laboratory: Challenges and Lessons Learned. *J Appl Lab Med*. 2021;6(5):1338-1354.	1, 3, 5
127. Uppal A, Silvestri DM, Siegler M, Natsui S, Boudourakis L, Salway RJ, et al. Critical Care And Emergency Department Response At The Epicenter Of The COVID-19 Pandemic. *Health Aff (Millwood)*. 2020;39(8):1443-1449.	1, 7, 8, 9
128. Van Nguyen H, Van Hoang M, Dao ATM, Nguyen HL, Van Nguyen T, Nguyen PT, et al. An adaptive model of health system organization and responses helped Vietnam to successfully halt the Covid-19 pandemic: What lessons can be learned from a resource-constrained country. *Int J Health Plann Manage*. 2020;35(5):988-992.	1, 6
129. Vorsters A, Bosch FX, Poljak M, Waheed DE, Stanley M, Garland SM; HPV Prevention and Control Board and the International Papillomavirus Society (IPVS). HPV prevention and control - The way forward. *Prev Med*. 2022;156:106960.	3, 10
130. Wade MJ, Lo Jacomo A, Armenise E, Brown MR, Bunce JT, Cameron GJ, et al. Understanding and managing uncertainty and variability for wastewater monitoring beyond the pandemic: Lessons learned from the United Kingdom national COVID-19 surveillance programmes. *J Hazard Mater*. 2022;424(Pt B):127456.	5
131. Waitzberg R, Triki N, Alroy-Preis S, Lotan T, Shiran L, Ash N. The Israeli Experience with the	1, 2, 3, 6, 10

"Green Pass" Policy Highlights Issues to Be Considered by Policymakers in Other Countries. *Int J Environ Res Public Health*. 2021;18(21):11212.	
132. Wang SSY, Teo WZY. Equitable and Holistic Public Health Measures During the Singaporean COVID-19 Pandemic. *Ann Glob Health*. 2021;87(1):45.	1, 3, 5, 6, 10
133. Williams MS, Myers AK, Patel VH, Marrast L, Maria NI, Marino J, et al. COVID-19 Conversations Within Black/Brown Minority Communities: A Stakeholder and Psychoeducation Approach Using Zoom/Facebook Live. *Health Promot Pract*. 2022;23(1):42-45.	3
134. Williams R, Bursac Z, Trepka MJ, Odom GJ. Lessons Learned From Miami-Dade County's COVID-19 Epidemic: Making Surveillance Data Accessible for Policy Makers. *J Public Health Manag Pract*. 2021;27(3):310-317.	2
135. Wilson AN, Sweet L, Vasilevski V, Hauck Y, Wynter K, Kuliukas L, et al. Australian women's experiences of receiving maternity care during the COVID-19 pandemic: A cross-sectional national survey. *Birth*. 2022;49(1):30-39.	8
136. Wong PW, Lam Y, Lau JS, Fok H. The Resilience of Social Service Providers and Families of Children With Autism or Development Delays During the COVID-19 Pandemic-A Community Case Study in Hong Kong. *Front Psychiatry*. 2021;11:561657.	1, 6, 8
137. Xu T, Sattar U. Conceptualizing COVID-19 and Public Panic with the Moderating Role of Media Use and Uncertainty in China: An Empirical Framework. H *Healthcare (Basel)*. 2020;8(3):249.	2
138. Zapata T, Buchan J, Azzopardi-Muscat N. The health workforce: Central to an effective response to the COVID-19 pandemic in the European Region. *Int J Health Plann Manage*. 2021;36(S1):9-13.	6,7, 9
139. Zhang H, Dimitrov D, Simpson L, Plaks N, Singh B, Penney S, et al. A Web-Based, Mobile-Responsive Application to Screen Health Care Workers for COVID-19 Symptoms: Rapid Design, Deployment, and Usage. *JMIR Form Res*. 2020;4(10):e19533.	3, 6, 7
140. Zhang T, Robin C, Cai S, Sawyer C, Rice W, Smith LE, et al. Public health information on COVID-19 for international travellers: lessons learned from a mixed-method evaluation. *Public Health*. 2021;193:116-123.	2, 4
141. Zhou J, Ghose B, Wang R, Wu R, Li Z, Huang R, et al. Health Perceptions and Misconceptions Regarding COVID-19 in China: Online Survey Study. *J Med Internet Res*. 2020;22(11):e21099.	1, 6, 7, 8
142. Zhou S, Han L, Liu P, Zheng ZJ. Global health governance for travel health: lessons learned from the coronavirus disease 2019 (COVID-19) outbreaks	2

in large cruise ships. *Glob Health J*. 2020;4(4):133-138.	
143. Zimmerman KO, Akinboyo IC, Brookhart MA, Boutzoukas AE, McGann KA, Smith MJ, et al. Incidence and Secondary Transmission of SARS-CoV-2 Infections in Schools. *Pediatrics*. 2021;147(4):e2020048090.	6
144. Zimmermann R, Sarma N, Thieme-Thörel D, Alpers K, Artelt T, Azouagh K, et al. COVID-19 Outbreaks in Settings With Precarious Housing Conditions in Germany: Challenges and Lessons Learned. *Front Public Health*. 2021;9:708694.	3, 6, 7, 9
